# The *tep1 *gene of *Sinorhizobium meliloti *coding for a putative transmembrane efflux protein and *N*-acetyl glucosamine affect *nod *gene expression and nodulation of alfalfa plants

**DOI:** 10.1186/1471-2180-9-17

**Published:** 2009-01-27

**Authors:** Pieter van Dillewijn, Juan Sanjuán, José Olivares, María José Soto

**Affiliations:** 1 Departamento de Protección Ambiental, Estación Experimental del Zaidín, CSIC, Profesor Albareda, 1, 18008 Granada, Spain; 2 Departamento de Microbiología del Suelo y Sistemas Simbióticos, Estación Experimental del Zaidín, CSIC, Profesor Albareda, 1, 18008 Granada, Spain

## Abstract

**Background:**

Soil bacteria collectively known as *Rhizobium*, characterized by their ability to establish beneficial symbiosis with legumes, share several common characteristics with pathogenic bacteria when infecting the host plant. Recently, it was demonstrated that a *fadD *mutant of *Sinorhizobium meliloti *is altered in the control of swarming, a type of co-ordinated movement previously associated with pathogenicity, and is also impaired in nodulation efficiency on alfalfa roots. In the phytopathogen *Xanthomonas campestris*, a *fadD *homolog (*rpfB*) forms part of a cluster of genes involved in the *r*egulation of *p*athogenicity *f*actors. In this work, we have investigated the role in swarming and symbiosis of SMc02161, a *S. meliloti fadD*-linked gene.

**Results:**

The SMc02161 locus in *S. meliloti *shows similarities with members of the Major Facilitator Superfamily (MFS) of transporters. A *S. meliloti *null-mutant shows increased sensitivity to chloramphenicol. This indication led us to rename the locus *tep1 *for *t*ransmembrane *e*fflux *p*rotein. The lack of *tep1 *does not affect the appearance of swarming motility. Interestingly, nodule formation efficiency on alfalfa plants is improved in the *tep1 *mutant during the first days of the interaction though *nod *gene expression is lower than in the wild type strain. Curiously, a *nodC *mutation or the addition of *N*-acetyl glucosamine to the wild type strain lead to similar reductions in *nod *gene expression as in the *tep1 *mutant. Moreover, aminosugar precursors of Nod factors inhibit nodulation.

**Conclusion:**

*tep1 *putatively encodes a transmembrane protein which can confer chloramphenicol resistance in *S. meliloti *by expelling the antibiotic outside the bacteria. The improved nodulation of alfalfa but reduced *nod *gene expression observed in the *tep1 *mutant suggests that Tep1 transports compounds which influence nodulation. In contrast to *Bradyrhizobium japonicum*, we show that in *S. meliloti *there is no feedback regulation of nodulation genes. Moreover, the Nod factor precursor, *N*-acetyl glucosamine reduces *nod *gene expression and nodulation efficiency when present at millimolar concentrations. A role for Tep1 in the efflux of Nod factor precursors could explain the phenotypes associated with *tep1 *inactivation.

## Background

The rhizobia-legume mutualistic symbiosis is characterized by the formation of root nodules in which the bacteria fix atmospheric nitrogen to generate nitrogen sources assimilable by the plant. Although the attack of phytopathogens on plants have a different outcome (i.e. disease), similar efficient strategies have been acquired by pathogenic and mutualistic bacteria to establish compatible associations with their host plants [1]. These include signals involved in cell-cell communication in bacterial populations but also in cross-kingdom communication with host plants [1].

Recently, swarming has been described in Rhizobiaceae [2,3]. This type of co-ordinated movement was previously associated with the virulence of pathogens. In *Sinorhizobium meliloti*, swarming motility was associated with the activity of a long-chain fatty acyl-CoA ligase (FadD) which upon disruption affected nodulation efficiency on alfalfa roots. The authors hypothesized that a fatty acid derivative dependent on FadD activity may act as an intracellular signal controlling motility and symbiotic factors. In fact RpfB, a close homolog of FadD in *Xanthomonas campestris *[4], is implicated in the synthesis of cis-11-methyl-2-dodecenoic acid, a low-molecular-mass diffusible signal factor (DSF) involved in the regulation of pathogenicity factors [5]. In *X. campestris *the homolog of FadD is surrounded by genes which also participate in several ways in the regulation of important virulence determinants [6]. Therefore, a closer look was taken at the genes of *S. meliloti *in the vicinity of the *fadD *locus to determine their participation in symbiosis and/or swarming. Of the putative genes in the neighbourhood, the ORF SMc02161 located upstream from *fadD *and transcribed divergently from this gene, shows significant identity to permeases of the Major Facilitator Superfamily (MFS) [7]. The MFS class of permeases is the second largest family of membrane transporters found, after the ABC transporters. Members of this protein superfamily are typically single-polypeptide secondary carriers, comprising of 10–14 transmembrane α-helices which are able to transport small solutes such as sugars or toxins in response to chemiosmotic ion gradients [7,8]. In this work, the role of SMc02161 in bacterial resistance to toxics, *nod *gene expression and nodulation of alfalfa is described.

## Results and discussion

### *S. meliloti *ORF Smc02161 potentially codes for a transmembrane transporter with striking homology to MFS permeases

To analyze the region surrounding the *fadD *gene of *S. meliloti*, the available sequence of *S. meliloti *1021 [9] was used. The analysis using BLAST [10] revealed an ORF (SMc02163) downstream of *fadD *with homology to phosphoglucose isomerase (*pgi*) while upstream a divergently coding ORF (SMc02161) showed high identity to permeases of the Major Facilitator Superfamily (MFS). In this study, we characterize specifically ORF SMc02161. Putatively, this ORF encodes for a 411 amino acid protein with 11 transmembrane motifs typical of inner membrane proteins. This protein has an ATP/GTP binding motif, an alanine rich region (PROSITE [11]) and has the multi-domain of the MFS that covers most of the protein (from amino acid 73 to 331). The product shows the highest identity (66%) with a putative MFS protein in *Beijerinckia indica *subsp. indica ATCC9039, and shares most identity to MFS related permeases, transmembrane proteins, sugar transporters and efflux proteins of bacteria belonging to the Rhizobiales and Burkholderiales orders. Unfortunately, the physiological functions of the closest SMc02161 homologs have not been experimentally tested. One of the few SMc02161 homologs with an experimentally assigned function is CmlR (P31141, 29% identity), a chloramphenicol resistance protein of *Streptomyces lividans *[12].

### The *S. meliloti *SMc02161 mutant shows higher sensitivity to chloramphenicol

**Figure 1:**
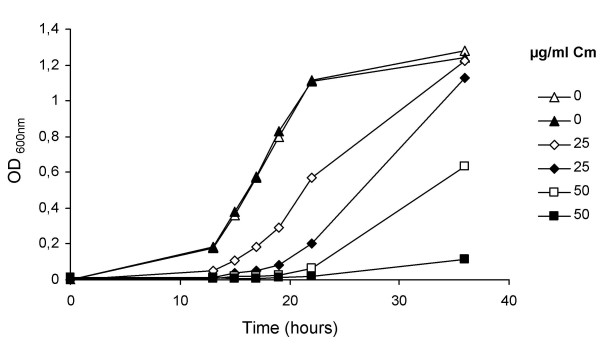
**Effect of different concentrations of chloramphenicol on the growth of *S. meliloti *GR4 and GR4T1**. Growth of GR4 (open symbols) and GR4T1 (*tep1 *mutant) (closed symbols) was tested in TY broth with 0 μg/ml (triangles), 25 μg/ml (diamonds) or 50 μg/ml (squares) chloramphenicol. A representative example from 3 independent experiments is shown.

To functionally characterize SMc02161, we constructed the GR4T1 mutant in which the wild type locus was replaced with a mutated version. Considering the homology shown by SMc02161 with CmlR, we compared the sensitivity of the GR4T1 mutant with the wild type *S. meliloti *strain GR4 to different concentrations of antimicrobial compounds such as chloramphenicol, tetracycline, and salicylic acid. The influence of luteolin and plant root exudates on the growth of these strains was also compared. Only the presence of chloramphenicol reduced the growth of the mutant compared to that of the wild type strain (Figure 1). This suggests that the protein encoded by SMc02161 can function as an efflux pump, expelling the antibiotic chloramphenicol from the bacteria. As a result, we renamed ORF SMc02161 to *tep1 *for *t*ransmembrane *e*fflux *p*rotein. To rule out possible polar effects of the created mutation in *tep1 *on downstream genes, complementation of the chloramphenicol sensitivity of the mutant was attempted with a plasmid construct. However, the results were inconclusive due to severe growth problems. Nevertheless, *tep1 *and the downstream gene of unknown function, SMc02160, have different expression patterns [13] and close homologs of these genes in other rhizobia are not located adjacently thereby suggesting that each form independent transcriptional units.

### *tep1 *is not necessary for swarming motility in *S. meliloti*

**Figure 2:**
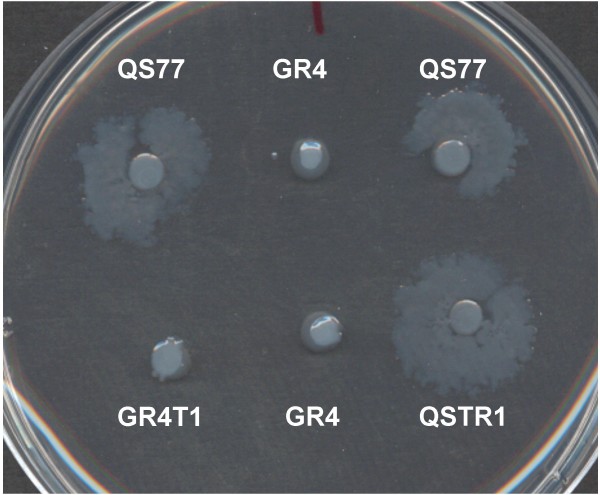
**Swarming motility of *S. meliloti *wild type and mutant strains**. Swarming motility of GR4 (wt), GR4T1 (*tep1 *mutant), QS77 (*fadD *mutant) and QSTR1 (double mutant *fadD*, *tep1*) was tested on 0.7% agar minimal medium at 28°C.

To determine if the function of *tep1 *is related to swarming as is the *fadD *product encoded upstream, swarming assays were performed. The results in Figure 2 show that the *fadD *mutant QS77 shows conditional swarming on semi-solid minimal medium (MM) plates containing 0.7% agar, in contrast to the wild type strain GR4. Likewise, the *tep1 *mutant GR4T1 does not show swarming. Furthermore, the *tep 1 *knock out mutant in a *fadD *mutant background, QSTR1, shows swarming as the *fadD *simple mutant, QS77 (Figure 2). Therefore, it appears that any substance possibly transported by *tep1 *is not involved in swarming motility.

### A *tep1 *mutation in *S. meliloti *improves nodule formation efficiency on alfalfa plants but shows reduced *nod *gene expression

**Figure 3:**
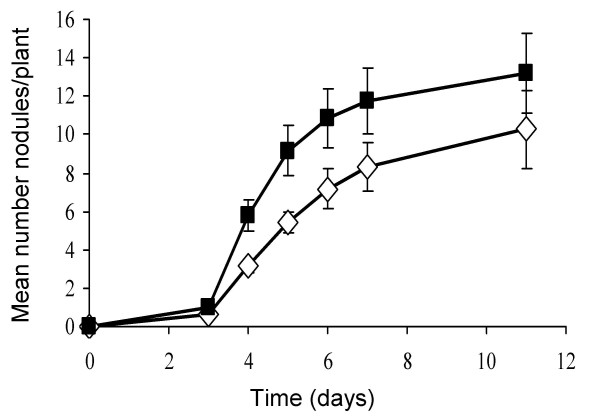
**Nodulation efficiency of *S. meliloti *GR4 (open diamonds) and GR4T1 (*tep1 *mutant) (closed squares)**. Mean values and standard errors (95% confidence) were calculated from three independent experiments.

To determine whether the activity of Tep1 is involved in symbiosis, the nodulation efficiency of the *tep1 *mutant was compared to the wild type strain. As shown in Figure 3, the mutant exhibits greater nodulation efficiency than the wild type strain during the first days of inoculation. Moreover, competition experiments in which alfalfa plants were co-inoculated with mixtures 1:1 of the wild type and mutant strains revealed that the lack of Tep1 confers a higher competitive ability to the bacterium (35% nodules occupied by the wild type strain *versus *49% nodules occupied by the *tep1 *mutant). These results suggest that Tep1 transports some type of compound which affects the nodulation of the host plant.

To check whether the greater nodule formation efficiency shown by the *tep1 *mutant correlates with an altered *nod *gene expression, activity of the *nodC*: *lacZ *fusion [14] was studied in the presence and absence of the inducer luteolin in either the mutant or wild type strain (Table 1). In contrast to the constitutively expressed promoter of the *npt *gene (P_km_::*lacZ*) [15], the induction of the *nodC::lacZ *transcriptional fusion in response to luteolin was reduced 3 fold in the *tep1 *mutant background compared to the *S. meliloti *wild type strain. This suggests that the product transported by Tep1 influences the luteolin-induction of the *nodC *gene. It is unlikely that lower uptake and/or accumulation of the flavonoid by the *tep1 *mutant is responsible for the observed effect. It has been reported that in *S. meliloti*, luteolin mostly accumulates in the outer membrane and only a relatively small amount of the flavonoid is present in the cytoplasmic membrane, in or on which the interaction with the NodD protein takes place [16]. It has been proposed that the accumulation of the flavonoid in the outer membrane protects the bacteria against the inhibitory effect of luteolin on NADH oxidase activity. As previously mentioned, we tested the effect of different concentrations (0, 5, 50 and 100 μM) of luteolin on the growth of the wild type and *tep1 *mutant strains. Although in both strains growth was negatively affected with increasing concentrations of the flavonoid, no differences could be detected (data not shown), suggesting that the mutation does not lead to different cellular concentrations of the inducer. Another possible explanation for the reduction of *nod *gene expression in a *tep1 *mutant would be that the mutation results in the accumulation of a compound which inhibits or interferes with the activation of the *nodC *promoter.

**Table 1:** Expression of transcriptional fusions to *lacZ *in *S. meliloti *GR4 and GR4T1.

		β-galactosidase activity (Miller U)	
		pGD499 (*npt::lacZ*)	pRmM57 (*nodC::lacZ*)
- luteolin	GR4	465 ± 38	47 ± 12
	GR4T1	435 ± 35	45 ± 14

+ luteolin	GR4	418 ± 34	777 ± 26
	GR4T1	398 ± 48	260 ± 45

β-galactosidase activity of the *npt::lacZ *and *nodC::lacZ *fusions were measured in the absence and presence of luteolin (5 μM). Mean values and standard errors (95% confidence) were calculated from three independent experiments.

### A *S. meliloti nodC *mutant is affected in *nod *gene expression

The results described above suggest that Tep1 transports a compound that has an effect on the number of nodules developed by the plant. The same or maybe a different compound transported by Tep1 also affects the induction of the *nodC *gene in response to luteolin. It is known that the strong, constitutive expression of the *nod *genes results in reduced nodulation phenotypes on legumes [17,18]. In *Bradyrhizobium japonicum *a feedback regulation of *nod *genes has been described [19]. The addition of chitin and lipochitin oligomers, or the expression of the β-glycosyl transferase NodC, reduces *nod *gene expression. These data together with the homology to sugar transporters shown by Tep1, prompted us to investigate whether the effects of the *tep1 *mutation could be due to alterations in the intra- and extracellular concentrations of Nod factors or Nod factor-related compounds. To our knowledge the existence or not of feedback regulation of *nod *genes in *S. meliloti *has not been investigated previously. Consequently, the expression of the *nodC *promoter was tested in GR4C5, a GR4-derivative *nodC *mutant, and compared with its activity in the *tep1 *mutant or in the wild type. The results (Table 2) show that in contrast to *B. japonicum *in which *nod *gene expression is elevated in a *nodC *mutant (1.6 fold) [19], *nod *gene expression is reduced 2.8 fold in the *S. meliloti nodC *mutant strain, reaching levels very similar to those shown by the *tep1 *mutant strain. This result indicates that in *S. meliloti *i) there is no feedback regulation of *nod *genes, and ii) a compound or compounds whose intracellular concentration is affected by the lack of NodC activity, interferes with *nod *gene induction. One of the most probable consequences of the lack of NodC activity is the accumulation of precursors of the Nod factor chitin backbone. To test whether changes in the concentration of these precursors could be responsible for the effects observed in the *nodC *and *tep1 *mutant, we decided to investigate how glucosamine and *N*-acetyl glucosamine influence both *nod *gene regulation in *S. meliloti *and nodulation of alfalfa plants.

**Table 2:** *nod *gene expression in *S. meliloti *GR4, the *tep1 *mutant and a *nodC *mutant.

Strain	β-galactosidase activity (Miller U)
GR4 (wt)	387 ± 48
GR4T1 (*tep1*)	144 ± 24
GR4C5 (*nodC*)	137 ± 34

β-galactosidase activity of the *nodC::lacZ *fusion was measured after bacteria had been incubated with 5 μM luteolin. Mean values and standard errors (95% confidence) were calculated from three independent experiments.

### Effect of glucosamine and *N*-acetyl glucosamine in *nod *gene expression in *S. meliloti *and on nodulation of alfalfa

To determine the possible role of core Nod factor precursors in *nod *gene regulation, studies were performed with glucosamine or *N*-acetyl glucosamine. The addition of glucosamine does not affect *nod *gene expression significantly in *S. meliloti *GR4 even when up to 50 mM glucosamine was added (data not shown). However, the addition of 5 mM *N*-acetly glucosamine reduces activity by more than 50% (Table 3). At higher concentrations (up to 50 mM) of *N*-acetly glucosamine the level of *nod *gene activity remains unchanged from that observed with 5 mM. Lower concentrations of the aminosugar (50 μM), only led to a slight reduction in *nodC *gene expression (data not shown). This indicates that in *S. meliloti *GR4, *N*-acetyl glucosamine can reduce *nod *gene expression.

**Table 3:** *nod *gene expression in *S. meliloti *GR4 with different concentrations of *N*-acetyl glucosamine.

mM NAGA	β-galactosidase activity (Miller U)
0	828 ± 251
5	425 ± 100
20	369 ± 112
50	412 ± 107

Expression of a *nodC::lacZ *fusion was measured in *S. meliloti *GR4 induced previously with 5 μM luteolin and different concentrations of *N*-acetyl glucosamine (NAGA). Mean values and standard errors (95% confidence) were calculated from three independent experiments.

**Figure 4:**
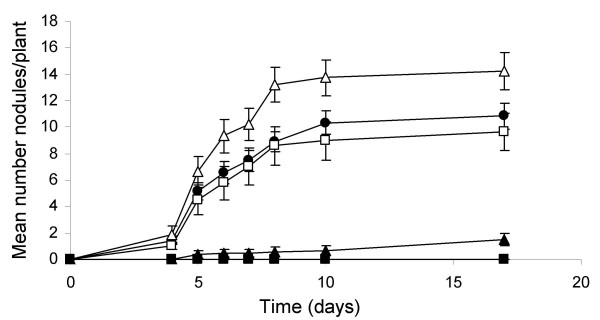
**Nodulation efficiency upon addition of different concentrations of Nod factor precursors**. Just before inoculation with *S. meliloti *GR4, alfalfa plants were supplemented with 50 μM glucosamine (GA) (open squares), 5 mM glucosamine (filled squares), 50 μM *N*-acetyl glucosamine (NAGA) (open triangles), 5 mM *N*-acetyl glucosamine (closed triangles) or without the addition of Nod factor precursors (filled circles). A representative example from 3 independent experiments is shown.

To determine if core Nod factor precursors also affect nodulation, the nodulation efficiency of alfalfa inoculated with *S. meliloti *GR4 was determined in the presence of different concentrations of glucosamine or *N*-acetyl glucosamine. The results in Figure 4 show that at the lowest concentration (50 μM) whereas glucosamine has no effect, *N*-acetyl glucosamine improves nodulation. It is known that *N*-acetyl glucosamines function as adhesins in some bacteria and that core Nod factor plays a role in biofilm formation in *S. meliloti*, facts that could explain the positive effect of the aminosugar on nodulation [20]. Surprisingly, the addition of 5 mM of glucosamine or *N*-acetyl glucosamine to the plant mineral solution, abolished or severely affected nodulation, respectively. As far as we know this is the first time that it has been shown that glucosamine or *N*-acetyl glucosamine inhibits nodulation by *S. meliloti*. The reason why these sugars at millimolar concentrations inhibit nodulation in alfalfa is not known but worth further investigation. We speculate that at high concentrations these compounds bind to and collapse plant lectins and/or Nod factor receptors interfering with the recognition of symbiotic bacterial signals. On the other hand, it is noteworthy that the effects of high concentrations of these Nod factor precursors on *nod *gene expression and nodulation are consistent with the effects observed in the *tep1 *mutant. Therefore, as a first attempt to correlate the presence of these compounds with Tep1 activity, we decided to investigate the effect of these aminosugars on *tep1 *transcription.

### Glucosamine and *N*-acetyl glucosamine activate *tep1 *transcription

Synthesis of transporters is often induced by the presence of their cognate substrates [21]. The expression of the *tep1 *gene was tested in *S. meliloti *GR4 harbouring pMPTR4 (*tep1::lacZ *transcriptional fusion) grown in different conditions. The results shown in Table 4 demonstrate that *tep1 *expression is higher in complex medium compared to defined minimal medium. Interestingly, the addition of glucosamine and *N*-acetyl glucosamine to the minimal medium increased transcription of *tep1*, suggesting that these aminosugars could be natural substrates of this putative transporter.

**Table 4:** *tep1 *gene expression in *S. meliloti *GR4 under different growth conditions.

Growth medium	β-galactosidase activity (Miller U)
TY	1523 ± 140
MM	449 ± 16
MM+GA	652 ± 33
MM+NAGA	792 ± 29

Expression of a *tep1::lacZ *fusion was measured in *S. meliloti *GR4 harbouring pMTR4 in TY medium, minimal medium (MM), and MM supplemented with either 5 mM glucosamine (GA) or 5 mM *N*-acetyl glucosamine (NAGA). Mean values and standard errors (95% confidence) were calculated from three independent experiments.

**Figure 5:**
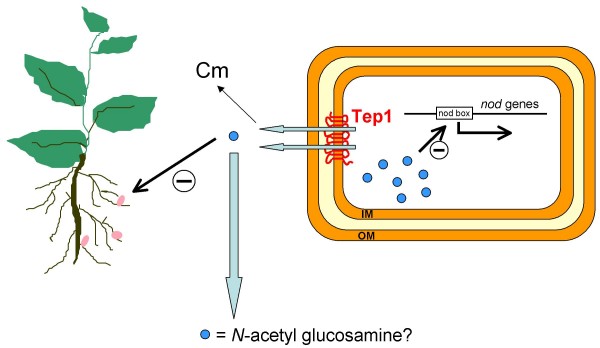
**Working model showing possible roles for Tep1 and their substrates**. Cm, chloramphenicol; IM, inner membrane; OM, outer membrane.

Considering all the results described here, we propose the following working hypothesis which is illustrated in Figure 5: Tep1 participates in the efflux of small compounds such as chloramphenicol and aminosugars which are core Nod factor precursors. Although these compounds have different structures, secondary multidrug (Mdr) transporters of the Major Facilitator Superfamily are known to be promiscuous in substrate recognition and transport [22]. In the *tep1 *mutant, chloramphenicol and Nod factor precursors accumulate inside the bacteria to concentrations which either hamper growth (chloramphenicol accumulation) or affect maximal *nod *gene expression (aminosugar accumulation). At the same time, the diminished efflux of aminosugars in the transport mutant leads to improved nodulation efficiency.

## Conclusion

The results obtained in this work suggest that the *tep1 *gene encodes a transport protein belonging to the MFS family of permeases able to confer chloramphenicol resistance in *S. meliloti *by expelling the antibiotic outside the cell. A *tep1*-linked gene in *S. meliloti*, *fadD*, plays a role in swarming motility and in nodule formation efficiency on alfalfa plants. We have demonstrated that *tep1 *is not involved in swarming motility but like *fadD *affects the establishment of the *S. meliloti*-alfalfa symbiosis. A *tep1 *loss-of-function mutation leads to increased nodule formation efficiency but reduced *nod *gene expression suggesting that Tep1 transports compounds which influence different steps of the nodule formation process. Whether these effects are caused by the same or different compounds putatively transported by Tep1, still needs to be investigated. Curiously, *nod *gene expression is reduced in a *S. meliloti nodC *mutant with the same intensity as in the *tep1 *mutant. This has implications for *nod *gene regulation in *S. meliloti *as it rules out the existence of a feedback regulation as described for *B. japonicum*. On the other hand, it could indicate that Tep1 is involved in the transport of Nod factors or its precursors. Indeed, increased concentrations of the core Nod factor precursor *N*-acetyl glucosamine reduced *nod *gene expression. Moreover, both glucosamine and *N*-acetyl glucosamine inhibit nodulation at high concentrations. Therefore, this constitutes the first work which attributes a role for core Nod factor precursors as regulators for nodulation of the host plant by *S. meliloti*. Furthermore, the results suggest that the activity of Tep1 can modulate the nodule formation efficiency of the bacteria by controlling the transport of core Nod factor precursors.

## Methods

### Bacterial strains, plasmids, media and chemicals

*Sinorhizobium meliloti *QS77 [2] is a *fadD*::Tn*5 *insertion mutant derivative of wild-type GR4 [23]. The plasmid pRmM57 (*nodC::lacZ *fusion) [14] was used to test the expression of the *nodC *gene and pGD499 (*npt::lacZ *fusion) [15] to test the expression of the constitutive kanamycin resistance gene. The pMPTR4 plasmid is a pMP220 [24] derivative in which an *Eco*RI fragment of 0.6 kb harbouring the intergenic *fadD*-*tep1 *region was cloned to create a *tep1::lacZ *transcriptional fusion. The pGUS3 plasmid containing an *nfeD::gusA *fusion was used in competition assays [25]. Triparental bacterial matings were performed using pRK2013 as helper plasmid [26]. *E. coli *was grown routinely at 37°C in Luria-Bertani medium (LB) [27]. *S. meliloti *strains were grown at 30°C in TY complex medium [28] or in defined minimal medium (MM) [29]. Growth was determined regularly in a spectrophotometer measuring the absorbance at 600 nm. Glucosamine and *N*-acetyl glucosamine were obtained from Sigma-Aldrich.

### Construction of a *S. meliloti tep1 *mutant

A null-mutant in ORF SMc02161 was obtained by allelic exchange. Firstly, a 3.6 kb *Sac*I fragment containing this ORF was subcloned from the *fadD *containing cosmid pRmersf442 [2] into pUC18 [30] to give pTrans1. To disrupt the ORF SMc02161 in pTrans1, a 2 kb *Sma*I fragment containing the streptomycin/spectinomycin resistance cassette from pHP45Ω [31] was inserted into a unique *Eco*RV site to give pTrans2. Next, the *Sac*I fragment containing the disrupted ORF was treated with T4 DNA polymerase (Roche Biochemicals) to make blunt ends and then cloned into the *Sma*I site of the suicide vector pK18*mobsac *[32] to give pTrans3. This vector was then used for allelic exchange by introducing it into the *S. meliloti *strains GR4, and the *fadD *mutant QS77 via triparental mating, and selecting putative mutants by streptomycin/spectinomycin resistance and sensitivity to sucrose. The resulting SMc02161 mutant GR4T1, and double *fadD*, SMc02161 mutant QSTR1 were confirmed by Southern hybridization with a specific probe.

### Construction of a *S. meliloti nodC *mutant

To obtain a *nodC *mutant in *S. meliloti*, a fragment was amplified from the chromosomal DNA of *S. meliloti *GR4 by PCR using 5'-CAGATTCAAGGTCACGAAGTGGCTAAC-3' and 5'-ATAAGCTTGTGACAGCCAGTCGCTATTG-3' as forward and reverse primers respectively. An *Eco*RI-*Pst*I fragment of 1.5 kb derived from the PCR product and containing half of the *nodB *gene and most of the *nodC *gene was subcloned into pUC18 [30] to obtain pGRC8. To disrupt *nodC*, pGRC8 was digested with *Sal*I and treated with Klenow (Roche Biochemicals) to create blunt ends. Next, the 2 kb *Sma*I fragment containing the streptomycin/spectinomycin resistance cassette from pHP45Ω [31] was introduced to give pNC150. The 3.5 kb *Eco*RI-*Pst*I fragment from pNC150 containing the disrupted *nodC *gene was inserted into *Eco*RI-*Pst*I digested pK18*mobsac *[32] to give pNC200. This suicide vector was then used to obtain the *S. meliloti nodC *mutant GR4C5, which was confirmed by Southern hybridization.

### Swarming behaviour assay

Swarming assays were performed as described previously [2]. Briefly, liquid cultures of *S. meliloti*, initiated from glycerol stocks, were grown at 30°C in TY broth with shaking to late logarithmic phase (optical density at 600 nm = 1–1.2). After incubation, cells were pelleted, washed twice in MM and resuspended in 0.1 volume of the latter medium. 2 μl drops of this suspension were deposited on the surface of plates containing MM with 0.7% agar and allowed to dry for 10 min. The plates were then inverted and incubated overnight (14–16 h) at 30°C and then scored for swarming motility.

### Plant assays

Alfalfa (*Medicago sativa *L.) seeds were sterilized and germinated as described by Olivares *et al*. [33]. To test the infectivity of the rhizobial strains, 24 individual plants were inoculated with each rhizobial suspension (10^6 ^colony forming units (cfu)/plant). To prepare the inoculants, rhizobial strains were previously grown in liquid TY medium up to an OD_600 _of 0.5 and then diluted accordingly. When addition of Nod factor precursors (glucosamine and *N*-acetyl glucosamine) was required, these compounds were added at the same moment as the bacterial inoculum. After inoculation, the number of nodulated plants and the number of nodules per plant were recorded daily.

To determine competitive ability, 12 plants were inoculated with GR4 × GR4 (pGUS3) or GR4T1 × GR4 (pGUS3) mixtures at ratios 1:1. The plasmid pGUS3 contains the marker gene coding for β-glucuronidase (GUS). To determine nodule occupancy, roots were collected 12 days after inoculation, briefly washed with water, and incubated overnight in the dark at 37°C in 1 mM X-Gluc (5-bromo-chloro-3-indolyl-β-D-glucuronide, Apollo Scientific, UK) in 50 mM sodium-phosphate buffer (pH 7.5) with 1% SDS. Those nodules occupied by GR4 (pGUS3) stain blue whereby nodule occupancy could be determined by counting blue and white nodules.

### Measurement of β-galactosidase activity

*S. meliloti *cells containing *lacZ *fusions were grown in liquid MM containing tetracycline to ensure plasmid maintenance. Bacteria were grown in liquid cultures overnight at 30°C to early logarithmic phase (OD_600 _of 0.2–0.4) in the presence or absence of 5 μM luteolin and different concentrations of glucosamine or *N*-acetyl glucosamine when required. Samples of 100 μl of the bacterial culture were taken and assayed for β-galactosidase activity by the SDS-chloroform method described by Miller [34].

## Authors' contributions

PvD performed experiments and wrote the manuscript, JS and JO helped coordinate the study, participated in its design and in the writing of the manuscript. MJS performed experiments, coordinated and designed the study and participated in the writing of the manuscript.
